# 

**Published:** 1891-07

**Authors:** 


### Southern Medical Record

A Monthly Journal of Medicine and Surgery.

VOL. XXI. ATLANTA, GA., JULY, 1891. NO. 7

EDITORS:

A. W. GRIGGS, M. D. FRANK O. STOCKTON, M. D.

WM. PERRIN NICOLSON, M D. DAN. H. HOWELL, M D., Bus. Mgr.

TERMS: $2.00 Per Annum; Single Copy, 20 Cents. Contents on Page 5.

Entered at the Postoffice at Atlanta, Ga., as Second-Class Matter. Index to Advertisements on Page 27

Postell & Sexton, Publishers, 47 S. Broad, Atlanta, Ga.

